# The association between glaucoma treatment adherence with disease progression and loss to follow-up

**DOI:** 10.1038/s41598-024-52800-2

**Published:** 2024-01-25

**Authors:** Laura Oltramari, Steven L. Mansberger, Júlia Mendonça Ponte Souza, Luciene Barbosa de Souza, Sarah Fumian Milward de Azevedo, Ricardo Y. Abe

**Affiliations:** 1 Hospital Oftalmológico de Brasília, Brasília, Brazil; 2 Devers Eye Institute at Legacy Health, Portland, OR USA; 3 Department of Ophthalmology, University of Campinas – UNICAMP, Campinas, Brazil

**Keywords:** Optic nerve diseases, Quality of life

## Abstract

Prospective cohort study from Brazil to evaluate glaucoma treatment adherence using a medication event monitoring system (MEMS) device and correlate with glaucoma progression and loss to follow-up (LTF) after one year of follow up. We included primary open glaucoma (POAG) patients treated with at least one ocular hypotensive eye drop. MEMS devices was used to monitor adherence for 60 days and evaluate the percentage of doses prescribed taken within the 60-day period. We classified patients according to rates of adherence: low adherence (less than 75% from MEMS measurements) and high adherence (more than 75% from MEMS measurements). We applied a questionnaire to investigated self-reported behavior towards treatment behaviors (glaucoma treatment compliance assessment tool, GTCAT). We also correlated rates of treatment adherence with clinical, demographical variables and the occurrence of glaucoma progression or LTF after one year of observation. We included 110 POAG patients and found that 28.18% of them were considered low adherent. We identify several variables associated with poor adherence such as glaucoma progression, LTF, younger age, low educational and income levels, absence of health insurance, years of disease and peak intraocular pressure. Several constructs from the self-reported GTCAT were also correlated with the rates of treatment adherence. To date, this is the first study in Latin America to evaluate glaucoma treatment adherence with MEMS devices and correlate adherence rates with glaucoma progression and LTF. We found a low-adherence rate of 28.18% and several additional risk factors were statistically associated with poor adherence.

## Introduction

Glaucoma is considered an optic neuropathy with a multifactorial cause, characterized by the loss of retinal ganglion cells, optic nerve atrophy and visual field loss^1^. Intraocular pressure (IOP) is the main risk factor for the progression of the disease, and although there are cases in which IOP is normal, it is the only factor at which we can interfere in order to slow down the damage to the optic nerve^2^. Despite many advances in treatment over the past two decades, glaucoma remains one of the leading causes of irreversible blindness worldwide^3^. As life expectancy increases, it is believed that there will be a significant increase in people with the condition around the world^4^. In Brazil, despite the low number of epidemiological studies, it is estimated that 3.4% of subjects over 40 years old may have glaucoma^5^. In addition, glaucoma seems to be important cause of visual impairment and blindness in some regions of Brazil^6^.

The clinical treatment of glaucoma aims to prevent the progression and avoid loss of quality of life due to visual impairment^7^. However, adherence to treatment in asymptomatic chronic diseases such as glaucoma is a delicate matter^8^. In addition, previous study has shown that around 30% of glaucoma patients can loss the follow-up (LTF) over a 10-year period and this can be associated with poor clinical outcomes, such as worsening of glaucoma^9^. Thus, investigating the relationship between LTF and treatment adherence is crucial.

Several barriers to treatment adherence were previously described, including forgetfulness, the large number of drugs prescribed, side-effects of medications, insufficient disease awareness about pathophysiology of glaucoma, absence of immediate benefits and inability to afford the medication^10,11^. Other studies have previously described adherence patterns to glaucoma treatment in Brazil^12–14^. However, none of those studies investigated rates of treatment adherence using objective metrics with monitoring devices and correlated adherence rates with glaucoma progression and LTF. Our study applied medication adherence monitoring system (MEMS) to objectively quantify rates of adherence and identify possible risk factors associated with poor adherence.

## Results

We included a total of 110 glaucoma participants. Table 1 shows a mean age of 68.9 years (± 9.77 years) with about half (57.2%) of the participants being female. 45.4% of the patients were Caucasian and 22.7% were African American. We found a mean adherence rate of 82.24 ± 19.7%. When considering the 75% cut-off for adherence, we estimated that 28.18% were non-adherent to treatment. Table 1 contains additional data including education and income. Around 75% of the patients were retired and 46.3% were married. 43.6% of the patients have health insurance. The mean time since glaucoma diagnosis (years of disease) was 6.7 years (± 8.3 years). 84.5% of patients reported that they instill the drops themselves, not requiring help from other people. The mean deviation from SAP was − 7.48 (± 7.44 dB) for the better eye and − 12.89 (± 9.60 dB) for the worse eye. Based on the mean deviation, patients were classified as having initial (32.7%), moderate (24.5%) and advanced (42.7%) glaucoma. From the total sample, 35 patients (33%) progressed (12 patients progressed exclusively from OCT, 10 patients progressed exclusively from SAP and 13 patients presented progression in both SAP and OCT). When analyzing only patients that presented progression during follow-up, per severity of glaucomatous damage, detection of progression exclusively by SAP was detected in 30% for initial, 10% for moderate and 60% for advanced glaucoma. Progression exclusively by OCT was detected in 33% for initial, 41% for moderate and 37% for advanced glaucoma. Finally, progression by SAP and OCT was detected in 28% for initial, 34% for moderate and 37% for advanced glaucoma. From the total sample, 23 patients (20.9%) presented LTF after one year of observation. Of these, approximately 14 patients were considered non-adherent to treatment. Of the 87 patients (79.0%) who maintained regular follow-up within the first year of observation, 65 of them were considered adherent to the treatment with eye drops.

**Table 1:** Clinical and Demographic Characteristics of Subjects of Study.

Parameters	Total subjects (n = 110)
Adherence rate %	
Mean + /− SD	82.24 + /− 19.76
Range	30.25 to 100
Age	
Mean + /− SD	68.91 + /− 9.77
Range	43 to 91
Gender	
Male	47 (42.73%)
Female	63 (57.27%)
Employment status	
Job	19 (17.27%)
Unemployed	10 (9.09%)
Retired	81 (73.64%)
Marital Status	
Married	51 (46.36%)
Single	17 (15.45%)
Widover	25 (22.73%)
Divorced	17 (15.45%)
Schooling	
Illiterate	11 (10.00%)
Elementary school incomplete	29 (26.36%)
Elementary school complete	17 (15.45%)
Middle school incomplete	2 (1.82%)
Middle school complete	18 (16.36%)
University incomplete	3 (2.73%)
University complete	30 (27.27%)
Race	
Caucasian	50 (45.45%)
African American	53 (51.73%)
Asian	4 (3.64%)
Health insurance	
Yes	48 (43.64%)
No	62 (56.36%)
Years of disease	
Mean + /− SD	6.73 + /- 8.34
Range	1 to 49
Who instill the eyedrop	
Patient	93 (84.55%)
Another person	17 (15.45%)
Mean Deviation	
Better eye	− 7.48 dB
Worst eye	− 12.89 dB
Glaucoma severity	
Initial	36 (32.73%)
Moderate	27 (24.55%)
Advanced	47 (42.73%)
Best corrected visual acuity	
Better eye	0.13
Worst eye	0.36

**Figure 1:**
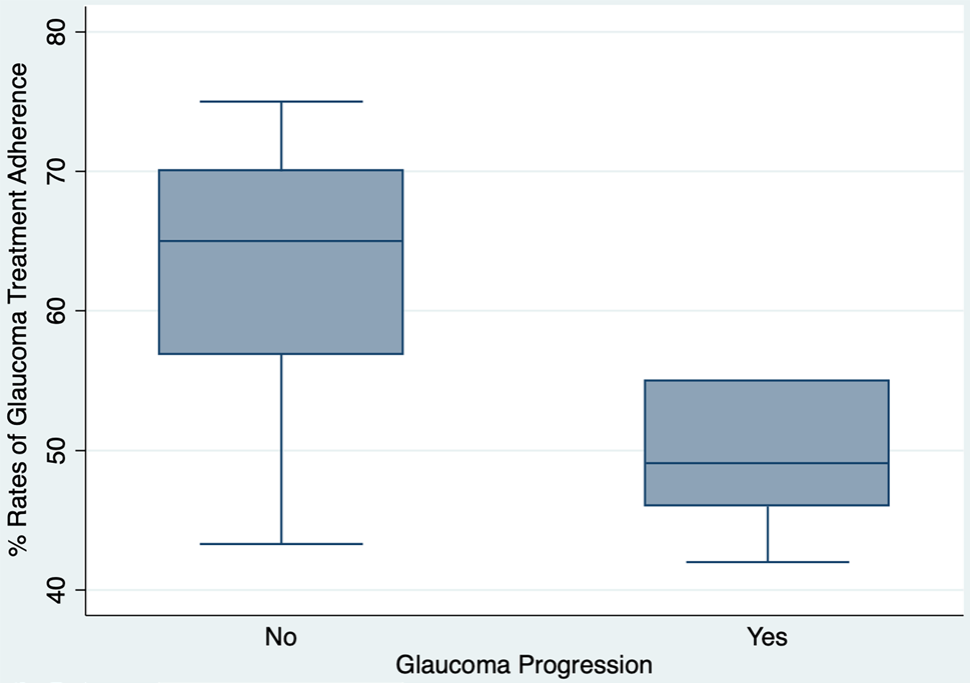
Box plot chart showing the rates of glaucoma treatment adherence according to glaucoma progression in patients in the low adherence group.

**Figure 2:**
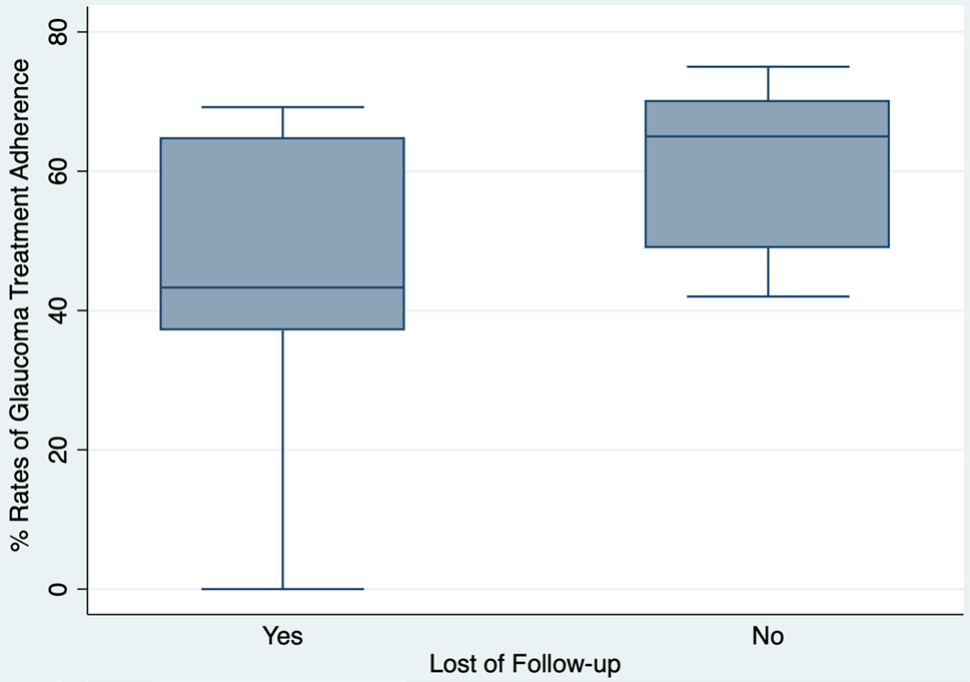
Box plot chart showing the rates of glaucoma treatment adherence according to loss of follow-up in patients in the low adherence group.

According to the Spearmen correlation analysis (Table 2), glaucoma progression was associated with the rates of adherence for both low adherence group (r = 0.42, *P = *0.049, Fig. 1) and high adherence group (r = 0.31, *P = *0.007). The rate of LTF was also correlated with adherence in low adherence group (r = 0.44, *P = *0.012, Fig. 2), but not with the high adherence group (r = 0.16, *P = *0.099). No other clinical demographical variable was related with adherence in the low adherence group. Whereas for patients with high adherence, several other variables were associated with poor adherence such as older age (r = 0.22, *P = *0.045), low educational (r = 0.31, *P = *0.005) and income (r = 0.24, *P = *0.033) levels, absence of health insurance (r = 0.38, *P < *0.001), years of disease (r = 0.23, *P = *0.004) and peak intraocular pressure (r = 0.26, *P = *0.020). We have also performed a univariable regression analysis and found that, LFT (*P = *0.006), education level (*P = *0.012), health insurance (*P = *0.018), years of disease (*P = *0.054) and visual acuity (*P = *0.040) were statistically correlated with rates of treatment adherence. However, In the multivariable analysis, none of the variables remained statistically significant (Supplemental Table).

**Table 2:** Correlation of different variables with rates of glaucoma treatment adherence.

Variable	Low Adherence (n = 22)		High Adherence (n = 88)	
	Correlation (rho)	*P* value	Correlation (rho)	*P* value
Glaucoma progression	0.42	0.049	0.31	0.007
Lost of follow-up	0.44	0.012	0.18	0.099
Age	0.10	0.561	0.22	0.045
Gender	0.27	0.139	0.16	0.157
Race	0.01	0.933	0.08	0.469
Marital status	0.13	0.478	0.08	0.452
Job status	0.24	0.183	0.04	0.688
Education level	0.04	0.923	0.31	0.005
Family income	0.07	0.671	0.24	0.033
Health insurance	0.31	0.079	0.38	< 0.001
Years of disease	0.29	0.103	0.23	0.004
Number of eye drops	0.04	0.981	0.06	0.551
Who instill the eye drop	0.03	0.835	0.10	0.087
Co-morbidity index	0.14	0.425	0.10	0.889
Better eye mean deviation	0.08	0.656	0.04	0.480
Worse eye mean deviation	0.03	0.843	0.08	0.966
Better eye visual acuity	0.02	0.980	0.14	0.119
Worse eye visual acuity	0.12	0.502	0.14	0.211
Peak intraocular pressure	0.24	0.186	0.26	0.020

We evaluated the responses from the GTCAT according to the constructs from the health belief model such as, knowledge on the severity of the disease, barriers associated with the lack of eyedrop, self-efficacy, side effect of treatment, susceptibility to the disease, cues to action and perception of the benefit of treatment. According to Table 3, for patients with low adherence, the construct of self-efficacy (r = 0.39, *P = *0.027) and cues to actions (r = 0.42, *P = *0.018) were associated with rates of adherence. For patients with high adherence, the constructs of knowledge on the severity of the disease (r = 0.39, *P < *0.001), barriers associated with the lack of eyedrop (0.43, *P < *0.001) and perception of the benefit of treatment (r = 0.27, *P = *0.15) were associated with rates of adherence.

**Table 3:** Correlation between rates of glaucoma treatment adherence with constructos from the Glaucoma treatment compliance assessment tool (GTCAT).

GTCAT constructs	Low adherence (n = 22)		High adherence (n = 88)	
	Correlation (rho)	*P* value	Correlation (rho)	*P* value
Knowledge on the severity of the disease	0.11	0.543	0.39	< 0.001
Barriers associated with the lack of eye drop	0.29	0.104	0.43	< 0.0001
Self efficacy	0.39	0.027	0.01	0.871
Side effect of treatment	0.142	0.445	0.14	0.217
Susceptibility to the disease	0.05	0.773	0.12	0.269
Cues to action	0.42	0.018	0.12	0.290
Perception of the benefits of treatment	0.16	0.389	0.27	0.015

GTCAT: Glaucoma treatment compliance assessment tool.

## Discussion

The adherence to glaucoma treatment is crucial to avoid the risk of progression of the disease, minimizing the chances of developing visual impairment and blindness. Studies that investigate the rates of adherence and identify possible risk factors for poor adherence are needed as the eyes drops remain as the mainstream option for glaucoma treatment, especially in underserved countries like Brazil, in which access to lasers and minimally invasive surgeries is difficult. To date, this is the first study in Latin America to apply electronic monitoring devices to objectively measure the rates of adherence of glaucoma patients. We have identified several barriers to adherence that can help us as clinicians to better guide our patients to improve their adherence to treatment.

We found a non-adherence rate of 28.18%, which corroborates the findings from previous studies in Brazil. Lopes et al. found a non-adherence rate of 32.3% and Castro et al. reported a rate of 20.2%, with both studies using questionnaires to measure the rates of adherence^12,13^. Silva et al. found a non-adherence rate of 20% applying structured interviews on 50 glaucoma patients^14^. It is important to note that even though these studies have not used objective measurements as ours, the rates of adherence are similar, showing that the average rate of proper adherence is lower than 70%. When comparing to other developing countries, we found that the non-adherence rate can exceeds 50%. In fact, a study carried out in a tertiary hospital in North India, 49% of the 150 patients interviewed reported problems in using glaucoma medications, with 16% of them reporting total non-compliance and 35% of patients demonstrated improper drop administration technique^15^. In another study conducted in a hospital in Hong Kong, of the 71 patients interviewed, 45 patients reported taking their medication incorrectly, with 44 patients (63.4%) admitting to having missed doses in the period between the last and the present follow-up visit^16^. Of the 44 patients, the majority (79.5%) reported missing a dose less than 5 times per week^17^. This low rate of adequate adherence and improper drop administration technique shows the importance of knowing our patient’s behavior towards the eyedrop administration.

In the current study, for both groups of patients (low and high adherence), the adherence rates were associated with glaucoma progression. Rossi et al., in a retrospective study that included 35 patients, with 36 months of follow-up showed that 25 (71.4%) patients with stable visual fields had a median adherence rate of 85%, while patients who presented visual field progression (n = 10, 28.6%) had a median adherence of 21%^17^. Newman-Casey et al. evaluated relationship between medication adherence and visual field progression in 306 subjects from the medication arm of the Collaborative Initial Glaucoma Treatment Study. Participants were followed up at 6-month intervals for up to 10 years. Self-reported medication adherence and visual fields were measured^18^. They found that worse medication adherence was associated with loss of mean deviation over time (*P = *0.005). In the current study, we have evaluated progression by SAP and OCT. We corroborate a previous study that investigated the odds of detecting progression between SAP versus OCT in different stages of glaucomatous damage, as our rates of progression showed that patients with early glaucoma can progress more frequently with OCT (initial 33% versus advanced 25%), whereas patients with advanced glaucoma can present progression more frequently with SAP (initial 30% versus advanced 60%)^19^. Our findings corroborate previous studies, that rates of treatment adherence can be associated with glaucoma progression. Although further studies with longer follow-up are necessary.

In the current study, we have set different time intervals for visits to define LTF according to the severity of glaucoma following the Preferred Practice Pattern from the American Academy of Ophthalmology, as rapid visual field progression may be detected earlier by performing more visual fields per year and the frequency of evaluations can be dependent of the severity of damage^20^. However, we acknowledge that, a shorter cut-off period for more advanced glaucoma patients could have created a larger percentage of LTF in those groups leading to biased results. On the other hand, as patients with advanced glaucoma are at higher risk of losing remaining visual field and more prone to lose visual-related quality of life, we consider a 4-month interval between visits an acceptable period for those patients. In fact, Davis et al. have reviewed 145,234 health records of patients that lost follow-up between 2007 and 2012 and identified 16 patients who had come to serious harm as a result of LTF, with a profound effect on their vision, which could have been prevented^21^. Interestingly, nearly all (88%) of the patients identified who came to serious harm had glaucoma. This highlights the fact that despite treatment and monitoring, patients with glaucoma still can become blind^22^. We found that patients with lower adherence rates to glaucoma treatment are also at higher risk to present LTF over time. Also, the component of cue-to-action from the GTCAT questionnaire were also correlated with low-adherence (Table 3), showing that those patients lack a stimulus to trigger the decision-making process to accept a recommended health action, such as using the eye-drops correctly or attending the ophthalmic consultations as requested.

We found that rates of adherence were associated with family income and educational level. In a study carried out in a tertiary hospital in New Delhi, from 150 glaucoma patients, only a fourth understood to some extent, the need for compliance, and the risk of progression to blindness if the drugs were not used^23^. Patients with postgraduate degree were more compliant with their medication (100%) in comparison to the less educated group (88.2%).In southern India, a study investigated 399 patients with newly diagnosed glaucoma. Patients with higher levels of education (30%) had an adequate follow-up, while only 18.5% of patients with primary education or no formal education had adequate follow-up^24^. Castel et al. approached 738 patients with glaucoma and assessed adherence using the medication possession ratio^25^. The study highlighted that, compared with good adherents, non-adherents subjects tended to be less educated (11 ± 4 years versus 12 ± 4 years of education, *P = *0.002), and have lower income (65% versus 53% declared earning an income below average, *P = *0.004). According to the latest survey carried out by the Brazilian Institute of Geography and Statistics, the proportion of people aged 25 or over who completed higher education in 2019 was only 17.4%. The rate of people aged between 55 to 64 with higher education in Brazil is 14.3%, whereas the world average is between 25 and 30%^26^. In Brazil, Magacho et al. have demonstrated that the monthly cost of glaucoma can correspond up to 15.5% of the familial income and around 45.2% of patients can present difficulty in buying their medications^27^. Thus, we should certify that glaucoma patients with lower level of education are able to understand the importance of using the eyedrops, as well as the benefits of proper treatment and consultations and assure that they have enough economic resources to acquire the medications.

We found that patients with lower levels of IOP had better adherence. In a study carried out in Italy with 56 patients evaluated for a period of 6 months using electronic monitoring device, no association between IOP and adherence was found, indicating that normal IOP does not necessarily imply good adherence^28^. Higher levels of IOP can lead doctors to assume a poor response to medication. In these cases, adding new drugs in attempt to achieve target IOP may be misleading as adherence rates tend to fall with more complex treatment regimens. Gray et al. evaluated the impact of individualized patient care on adherence separating patients in two groups^29^. The intervention group were assigned to an intervention nurse, who conducted face-to-face needs assessments for more than 24 months. Interestingly, improved adherence did not lead to a statistically significant difference in IOP after 12 months, with mean IOP in the intervention group was 16.9, (± 3.6 mmHg) versus 17.4, (± 3.5 mmHg) for the control group^26^. Despite our finding that patients with lower levels of IOP had better adherence rates, no causality effect between these two variables can be established. In the current study, we found significant correlation in the Spearmen analysis and univariable linear regression analysis, whereas in the multivariable no correlation was found between variables. This highlights the fact that adherence in glaucoma treatment is a multifactorial and complex issue that can be related to multiple variables. However, we do not believe that this invalidate our results, as we were still able to show correlation in the Spearmen correlation and in univariable analysis, which suggest a trend in which future studies could further investigate.

In patients with high adherence, we found that patients with older age had worse rates of treatment adherence (r = 0.22, *P = *0.045). Friedman et al. have previously reported that patients with less than 50 years or more than 80 years of age are more like to have poor adherence^30^. In the current study the median age was 70 years (IQR: 62–75 years), which could explain the fact that correlation was stronger in older patients, as we had a small number of patients below 50 years old. The current study found that patients with health insurance had better rates of adherence. In Brazil it is estimated that only 25% of the population have access to private health insurance and these rates can vary in different regions of Brazil. In the current study more than 45% of our sample had health insurance. Thus, we could imply that in Brazil, in general, we would find even lower levels of adherence than reported. The present study found that patients with more years of the disease presented better adherence rates. In fact, Friedman et al. found that patients who have undergone anti-glaucoma therapy for less than a year are less compliant^30^. In their study, 32 patients started using eyedrops less than a year with a non-adherence rate of 48.4%, and 162 patients were undergoing treatment for more than a year with a non-adherence rate of 43.8%. A cross-sectional study carried out in Brazil with 255 glaucoma patients used the Morisky Adherence Scale to assess adherence to treatment^31^. Patients newly diagnosed with glaucoma (less than five years) comprised only 34.1% of the study sample but corresponded to 63.3% of the individuals who interrupted their glaucoma treatment (*P = *0.001). In the current study we chose not to include newly diagnosed glaucoma patients.

The GTCAT has been widely used to assess adherence in glaucoma patients due to its good construct validity with proper Rasch psychometric properties and containing specific constructs from the Health Belief Model that health care providers may address to improve adherence^32,33^. In the current study, in patients with low adherence, the GTCAT constructs of self-efficacy (r = 0.39, *P = *0.027) and cues to actions (r = 0.42, *P = *0.018) were associated with rates of adherence. For patients with high adherence, the constructs of knowledge on the severity of the disease (r = 0.39, *P < *0.001), barriers associated with the lack of eyedrop (0.43, *P < *0.001) and perception of the benefit of treatment (r = 0.27, *P = *0.15) were associated with rates of adherence. It is important to investigate the performance of self-reported adherence questionnaires as objective measurements with electronic monitoring devices in clinical practice is not always feasible, due to the cost of the devices and not all patients may agree to be electronically monitored. Hopefully future studies may investigate whether specific questions from the GTCAT could eventually serve as a biomarker for showing those with patient at higher risk for poor adherence.

This study has several limitations. First, although we reported the around 81% of patients were adherent go treatment, we did not assess instillation technique to evaluate whether patients were using the medication properly. Second, although we have included patients from 2 different glaucoma centers to recruit individuals with different degrees of socio-economic and health insurance status, Brazil has very distinct socio-economic differences according to regions, and future multicentric studies should be performed to validate our results. Third, IOP was assessed only using measurements at the inclusion visit. Future studies can include longitudinal IOP assessments to identify if IOP fluctuations can be related with adherence. Fourth, patients under clinical studies monitored with devices can present higher rates of adherence. Even though patients stayed 60 days with the MEMS devices, which could have minimized this effect, we can imply that the actual rates of true adherence can be lower than we have reported for patients in a real-world scenario. Fifth, glaucoma is a chronic and insidious disease and evaluating progression for just one year might not be enough, however even for this small interval of the study (1 year), we were able to detect patients that presented progression (35 patients), suggesting that if a longer monitoring was performed a higher number of patients could have presented progression. The uncommon number of patients that progressed under treatment can be related to the fact that we included patients without health insurance, which could lead to worse follow-up delaying the detection of IOP spikes or inability to afford with the eye-drops, leading to glaucomatous progression. Finally, during the follow-up, the interval between visits were different according to the stage of the disease, which could have increased the difficulty to detect changes in patients with initial glaucomatous damage, as these patients had less visits compared to patients with moderate and advanced disease. However, we evaluated both functional and structural measurements, using both SAP visual field parameters and OCT scans, improving the chances of progression detection as retinal nerve fiber layer evaluation can occurs more commonly in patients with mild stage of disease, comparing to those in later stages of the disease. In the current study, we have not included newly diagnosed glaucoma patients. Thus, all patients already had proper baseline visual field and OCT evaluation, which made the detection of progression more reliable. In addition, from the total of 35 patients that presented progression, 25 patients (72%) had moderate or advanced glaucoma, with 2–3 visits during the one-year follow-up.

The inappropriate use of eye drops may increase the inadvertent use of health resources, resulting in a reduction of effectiveness over treatment and an increased risk of treatment failure. In addition, it generates the need for excessive medical appointments and complementary exams, unjustified increases in doses, waste of pharmaceutical supplies and may increase the chances of the patient present progression of the disease ^31,32^. In conclusion, this is the first study in Latin America to objectively measure the rates of adherence of glaucoma patients using electronic monitoring devices. We found that the rates of adherence to glaucoma treatment were statistically correlated with glaucoma progression and LTF.

## Materials and methods

### Patients’ recruitment

This prospective cohort study included primary open angle glaucoma (POAG) patients from the Department of Glaucoma at the Hospital Oftalmológico de Brasília (HOB) and the Fundação Regional de Assistência Oftalmológica (FRAO), Brasília, Brazil. The institutional review board at the HOB approved the methods, and we obtained written informed consent from all participants. All study methods complied with the Declaration of Helsinki guidelines for human subject research (IRB: 3479310.6.0000.5667). All patients underwent a complete ophthalmological examination (visual acuity, slit-lamp biomicroscopy, intraocular pressure measurement, gonioscopy with Possner goniolens, dilated fundoscopic examination and optic disc photography) and completed a socio-demographic form and the Glaucoma Treatment Compliance Assessment Tool (GTCAT).

Subjects underwent standard automated perimetry (SAP) using the 24-2 Swedish interactive threshold algorithm (Carl Zeiss Meditec, Inc, Dublin, CA). To be included, subjects had to have a diagnosis of POAG using at least one ocular hypotensive medication. Glaucoma diagnosis was based on the presence of repeatable (at least two consecutive) abnormal SAP results with corresponding evidence of glaucomatous optic neuropathy in at least one eye. An abnormal SAP result was defined as a pattern standard deviation with *P < *0.05, and/or Glaucoma Hemifield Test results outside normal limits. In some patients we also used measurement of retinal nerve fiber layer from optical coherence tomography (OCT) to confirm diagnosis (Avantis, Optovue, Fremont, CA)^34^. Patients with angle closure glaucoma and secondary glaucomas were not included. Newly diagnosed glaucoma patients were not included since treatment patterns could vary along the first months of the disease leading to bias when comparing to patients with more time of disease. After measuring the adherence rates over the 2-month period, patients were monitored for 1 year to detect glaucoma progression (with SAP, retinography and OCT imaging) and/or loss to follow-up (LTF).

The Hodapp, Parish and Anderson classification system was used to classify the glaucomatous damage^35^. Previous studies have shown that disease progression in some patients may be detected first as either structural deterioration or worsening visual field^19^. Because the relationship between baseline disease classification and the detection of progression by structural and functional is not, well established, we applied both tests simultaneously to monitor progression, with the clinicians (SFMA and RYA) masked to the measurement rates from MEMS caps^36^. We define progression using functional assessment with SAP visual field using both trend and event analysis (Humphrey Glaucoma Progression Analysis)^37^. We also evaluated structural changes with OCT retinal nerve fiber layer analysis using Avantis (Optovue) software to detect changes applying both event (comparing thickness and deviation maps) and trend analysis (linear regression over time from the software)^38^. All exams were evaluated by two glaucoma specialist (RYA and SFMA) to assure structure–function correlations and also agreement between OCT scans with retinography and fundus examination. Both exams (visual fields and OCT imaging) were performed in the visits that occurred according to the severity of the disease (according to the severity of glaucoma, as follow: 9 months for initial, 6 months for moderate and 4 months for advanced glaucoma). Our national primary open angle glaucoma consensus (from the Brazilian Glaucoma Society) has defined to monitor glaucoma patients according to the severity of the disease, suggesting a 4-month interval between visits for patients with advanced glaucoma according to Hodapp and Parish visual field classification. Kim et al. defined LTF as greater than 12 months after proposed follow-up^39^. However due to the diversity of glaucoma damage of our sample, we consider LTF if the patient did not showed to consultation after successive clinic contacts (according to the severity of glaucoma, as follow: 9 months for initial, 6 months for moderate and 4 months for advanced glaucoma)^40^.

### Glaucoma treatment compliance assessment tool (GTCAT)

The GTCAT was developed by Mansberger et al. using constructs of the Health Belief Model, expert opinion, and previous studies regarding adherence in glaucoma patients^41,42^. The GTCAT consists of a 27-item form, of which 3 items are aimed at identifying barriers associated with treatment, 6 items related to forgetting due to lack of cues-to-action component, 2 items with stimulus or cues for actions, 7 items related to self-knowledge about the disease, 1 items address the doctor-patient relationship, 1 item addresses the perception of adherence to treatment, 3 items address knowledge on the severity of the disease and 3 items address the patient's susceptibility to the disease. This questionnaire was previously translated into Brazilian Portuguese, and was psychometric validated with Rasch analysis^33^.

### Socio-demographic form

Data were collected in relation to age, gender, race, marital and job status, education and income level, presence of health insurance, years of glaucoma diagnosis, number of hypotensive eyedrops, who instill the eyedrop. We also reviewed medical history for any of the following co-morbidities: diabetes mellitus, arthritis, hypertension, heart disease, depression, asthma, and cancer. A simple summation score was used to generate a co-morbidity index^43^.

### Medication event monitoring system (MEMS)

Medication adherence to glaucoma was assessed with a bottle equipped with a medication adherence monitoring system (MEMS). This system contains a microchip that records the date and time when the patient opens and closes the bottle. Previous studies have reported that up to 30% loss of follow-up can occur if patients stay with the MEMS devices for a 3-month period^44,45^. Thus, data from the devices were extracted at the patient's return 60 days (approximately) from the date of delivery, where the device was returned to the researcher.

The researcher trained all participants to place the eye drops into the monitoring device as soon as the patient arrived home from the initial visit, and to open it only when the medication was to be instilled in the eyes, and to close the bottle after use. To avoid test openings by the patient or researcher, the first day on which the patient started using the device was excluded, as well as the day of the final visit on which the patient returned the device to the researcher. AARDEX Power View software (version 3.5.1; Mead Westvaco Ltd, Sion, Valais, Switzerland) was used to analyze the time each bottle was unscrewed, and the total number of openings performed.

For patients who use the medication once a day (prostaglandins), a "valid opening" was counted as the one that occurred within 24 h (2 h more or less), that is, they were not counted as "valid opening” those that occurred less than 22 h apart or more than 26 h apart. For patients who use a medication twice a day, those that occurred within 12 h (2 h more or less) were counted as "valid opening", that is, they were not counted as "valid opening" those that occurred less than 10 h apart or more than 14 h apart. The classes of antihypertensive eye drops evaluated were prostaglandin analogues, carbonic anhydrase enzyme inhibitors, alpha2-agonists and beta-blockers. Each eyedrop had a bottle with an exclusive monitoring system for its use, with a maximum of two devices being delivered to each patient. We evaluate the percentage of doses prescribed taken within the 60-day period. For example, if a patient were prescribed a prostaglandin, then the bottle should have been unscrewed 60 times over a 60-day period. If the patient only unscrews the bottle 45 times, then their percent adherence would be 45 divided by 60 and multiplied by 100% (or 75%). In addition to that, we also calculated the number of invalid openings (if the patient used the medication more than 2 h than expected). For example, if the patient using a prostaglandin monotherapy, unscrew the bottle 50 times and presented 5 invalid openings, then their percent adherence would be 45 divided by 60 and multiplied by 100% (or 75%). The patient was considered “non-adherent” when these parameters was below 75%. This definition of a non-adherent patient is empirical and based on previous work that has been carried out in this area^46^. For participants using more than one drug, we calculated the percentage of adherence separately for each MEMS device and then we average the results by the mean value from both devices, following the same criteria previously described.

### Statistical analysis

The pattern of glaucoma treatment adherence is not normally distributed and studies about adherence should not be based exclusively on parametric analysis^47^. In fact, Jones et al. have previously suggested that investigations should be performed separately for different range of adherence rates^47^. In the current study, we have divided patients according to rates of adherence: low adherence (less than 75% from MEMS measurements) and high adherence (more than 75% from MEMS measurements), based on previous study^48^. We performed Spearman rank correlation (nonparametric measure) to correlate the rates of adherence with glaucoma progression, loss of follow-up, clinical and demographical variables and also constructs from the self-reported GTCAT. We estimated that at least 100 patients would be required to achieve 80% power for a two-tailed test of Spearman's coefficient at significance level 0.05, considering an estimated correlation of 0.3 between adherence and progression rates. In addition to that, our sample size is in accordance with previous studies from Sanchez et al. ^27^ Since glaucoma treatment adherence is complex and multifactorial subject, we have also performed a univariable and multivariable regression analysis between the rates of treatment adherence and other variables, including in the multivariable model only variables with *P < *0.100. All statistical analyses were conducted with STATA, version 13 (StataCorp LP, College Station, Texas, USA). The alpha level (type I error) was set at 0.05.

### Supplementary Information

Supplementary Table 1.

## Data Availability

The datasets used and/or analyzed during the current study are available from the corresponding author on reasonable request.
