# Supplementary material for: The association between glaucoma treatment adherence with disease progression and loss to follow-up
Source: Sci Rep. 2024 Jan 25;14:2195. doi: 10.1038/s41598-024-52800-2 (PMC10810888; doi:10.1038/s41598-024-52800-2)
Supplement: Supplementary file 1 — Supplementary Table 1. [file 41598_2024_52800_MOESM1_ESM.docx]

**Supplemental Table.** Univariable and multivariable correlation between different variables with rates of glaucoma treatment adherence.

|  | **Univariable** | |  | **Multivariable** | |
| --- | --- | --- | --- | --- | --- |
|  |  |  |  |  |  |
| **Variable** | **Coefficient** | **P value** |  | **Coefficient** | **P value** |
| Glaucoma progression | 2.56 | 0.473 |  | N.A | N.A |
| Lost of follow-up | 12.50 | 0.006 |  | 7.67 | 0.120 |
| Age | 0.19 | 0.309 |  | N.A | N.A |
| Gender | 4.42 | 0.248 |  | N.A | N.A |
| Race (African American, yes) | 1.45 | 0.339 |  | N.A | N.A |
| Marital status (single, yes) | 2.58 | 0.525 |  | N.A | N.A |
| Job status (retired, yes) | 5.75 | 0.346 |  | N.A | N.A |
| Education level (college degree, yes) | 11.4 | 0.012 |  | 8.25 | 0.105 |
| Family income (higher income, yes) | 4.72 | 0.267 |  | N.A | N.A |
| Health insurance | ´-4.84 | 0.018 |  | ´-6.40 | 0.259 |
| Years of disease | 0.43 | 0.054 |  | 0.28 | 0.203 |
| Number of eye drops | 3.95 | 0.145 |  | N.A | N.A |
| Who instill the eye drop | 8.03 | 0.124 |  | N.A | N.A |
| Co-morbidity index | 1.85 | 0.431 |  | N.A | N.A |
| Better eye mean deviation | 2.79 | 0.260 |  | N.A | N.A |
| Worse eye mean deviation | 1.05 | 0.580 |  | N.A | N.A |
| Better eye visual acuity | ´-16.19 | 0.040 |  | ´-9.24 | 0.232 |
| Worse eye visual acuity | ´-11.23 | 0.002 |  | N.A | N.A |
| Peak intraocular pressure | 0.514 | 0.044 |  | N.A | N.A |
